# Imaging of Intracellular and Plasma Membrane Pools of PI(4,5)P_2_ and PI4P in Human Platelets

**DOI:** 10.3390/life11121331

**Published:** 2021-12-01

**Authors:** Ana Bura, Antonija Jurak Begonja

**Affiliations:** Department of Biotechnology, University of Rijeka, 51000 Rijeka, Croatia; ana.bura@biotech.uniri.hr

**Keywords:** platelets, phosphatidylinositol-4,5-bisphosphate, phosphatidylinositol-4-monophosphate, immunofluorescence, plasma membrane

## Abstract

Phosphoinositides (PIs) are phosphorylated membrane lipids that have a plethora of roles in the cell, including vesicle trafficking, signaling, and actin reorganization. The most abundant PIs in the cell are phosphatidylinositol-4,5-bisphosphate [PI(4,5)P_2_] and phosphatidylinositol-4-monophosphate (PI4P). The localization and roles of both PI(4,5)P_2_ and PI4P are well established, is the broadly accepted methodological approach for their immunocytochemical visualization in different cell compartments in several cell lines. However, not much is known about these PIs in platelets (PLTs), the smallest blood cells that detect vessel wall injury, activate, and stop the bleeding. Therefore, we sought to investigate the localization of PI(4,5)P_2_ and PI4P in resting and activated PLTs by antibody staining. Here, we show that the intracellular pools of PI(4,5)P_2_ and PI4P can be detected by the established staining protocol, and these pools can be modulated by inhibitors of OCRL phosphatase and PI4KIIIα kinase. However, although resting PLTs readily stain for the plasma membrane (PM) pools of PI(4,5)P_2_ and PI4P, just a few activated cells were stained with the established protocol. We show that optimized protocol allows for the visualization of PI(4,5)P_2_ and PI4P at PM in activated PLTs, which could also be modulated by OCRL and PI4KIIIα inhibitors. We conclude that PI(4,5)P_2_ and PI4P are more sensitive to lipid extraction by permeabilizing agents in activated than in resting human PLTs, which suggests their different roles during PLT activation.

## 1. Introduction

Platelets (PLTs) are the smallest blood cells (2–3 µm in diameter) that form from the biggest cells in the bone marrow, megakaryocytes (MKs). PLTs detect vessel wall injury, change shape, adhere to the site of injury, and aggregate to form a clot. In addition to this primary function in hemostasis and thrombosis, PLTs are also a part of innate immunity and have a role in inflammatory reaction, inflammatory diseases, and the regulation of angiogenesis [1,2,3]. In their resting state, they have a discoid shape. Once they activate, they undergo extensive actin reorganization accompanied by shape change. During spreading, PLTs first become rounded or spheroid, followed by the formation of fingerlike projections, called filopodia, that originate from the plasma membrane (PM). Finally, the surface of the PLTs flattens, lamellae are formed, granules and organelles are moved to the center of the cell, and the cells form the so-called “fried egg” appearance [4].

Membrane remodeling and vesicular trafficking are needed for PLT activation, and these processes are mediated by phosphoinositides (PIs), phosphorylated membrane lipids. Two abundant PIs in the cell are phosphatidylinositol-4,5-bisphosphate [PI(4,5)P_2_] and phosphatidylinositol-4-monophosphate (PI4P) [5]. PI(4,5)P_2_ is mostly found on the PM, where it acts as a signaling molecule [6,7]. At the PM, it can be hydrolyzed by protein lipase C (PLC), which results in the formation of second messengers, diacylglycerol (DAG) and inositol-1,4,5-trisphosphate (IP3) [8]. PI(4,5)P_2_ also recruits proteins important for the function of actin cytoskeleton like Wiskott-Aldrich syndrome protein (WASP) and profilin as well as adaptor proteins for the formation of clathrin-coated vesicles (CCVs) [9]. PI4P is mostly found at the Golgi apparatus and the PM, but it can also be found on the endoplasmic reticulum (ER) [5] and cytoplasmic vesicles, such as late endosomes and lysosomes [10]. At the Golgi apparatus, PI4P recruits clathrin adaptors and Golgi-localized, Gamma-adaptin ear homology, Arf-binding proteins (GGAs) [11], and it has a role in the regulation of *N*- and *O*-glycosylation [12]. On the PM, PI4P can serve as a substrate for the formation of PI(4,5)P_2_ or it can contribute to the polyanionic lipid pool that defines the inner leaflet of the PM [13].

Localization of PIs within cells can be visualized by molecular probes expressed from plasmids or by antibody staining. Molecular probes consist of protein domains that specifically bind certain PIs and allow imagining in live cells [14]. However, anucleate PLTs cannot be transfected, therefore the only way to visualize PIs is by antibodies. It has been shown in different cell lines that, depending on the immunocytochemical technique, PI(4,5)P_2_ and PI4P can be visualized at different intracellular compartments. If the staining is performed at room temperature with the permeabilizing agent digitonin, intracellular pools of PIs will be detected [15]; digitonin replaces cholesterol-lipid contacts in the membrane, forming permanent pores, while room temperature allows deep penetration of the permeabilizing agent [16]. In contrast, if the staining is performed on ice with permeabilizing agent saponin, the PM pool of PIs will be detected [15]; saponin selectively binds cholesterol, resulting in the formation of reversible holes in the PM, while ice-cold temperature, lowering the movements of molecules, does not allow deep penetration of the permeabilizing agent or antibodies [17].

Since in PLTs both vesicular trafficking and membrane reorganization are important parts of their activation, we wanted to visualize PI(4,5)P_2_ and PI4P in intracellular compartments as well as on the PM in both resting and activated PLTs. We questioned whether we could visualize these two PI pools by immunofluorescence, if their levels could be modulated by inhibitors of PI kinases and phosphatases, and if the localization would differ in resting and activated states. Here, we show that in resting and activated PLTs, both PI(4,5)P_2_ and PI4P localize to intracellular compartments and that their levels can be modulated by inhibiting OCRL, a phosphatase that dephosphorylates PI(4,5)P_2_, resulting in the formation of PI4P, or inhibiting PI4KIIIα, a major kinase that produces PI4P mostly at the PM. With previously established protocols, PI(4,5)P_2_ and PI4P could be readily detected in HEK293T and BALB3T3 cells, as well as intracellular pools of PIs in PLTs. However, the staining of PIs at PM was ineffective, mostly in activated PLTs. Therefore, we tested several conditions and modified the staining protocol to detect both PI(4,5)P_2_ and PI4P in PM of activated PLTs. We conclude that PI(4,5)P_2_ and PI4P are more sensitive to lipid extraction or antibody recognition in activated compared to resting human PLTs, which could reflect different PLT needs and functions for these two PIs in both states.

## 2. Materials and Methods

### 2.1. Antibodies

Antibodies were obtained from the following resources: monoclonal mouse anti-PI(4,5)P_2_ (Z-P045) and anti-PI4P (Z-P004), both IgM, were from Echelon Biosciences, polyclonal rabbit CD42b/anti-GPIbα was from Novus Biological (NBP2-89128), phalloidin (A12379) conjugated with Alexa Fluor (AF)-488 was from Invitrogen, monoclonal mouse IgM (MAB1326, R&D Systems,, Abigdon, UK) was a kind gift from Dr. Jelena Ban, (Laboratory of Molecular Neurobiology, Department of Biotechnology, University of Rijeka), polyclonal rabbit α-tubulin (SAB4500087, Sigma Aldrich, Taufkirchen, Germany) was a kind gift from Dr. Iva Tolić (Laboratory of Cell Biophysics, Ruđer Bošković Institute), and DAPI (D9542) was from Sigma Aldrich. Secondary antibody goat anti-mouse conjugated with Alexa Fluor (AF)-568 was from Invitrogen.

### 2.2. Human Platelet Isolation and Platelet Spreading

PLTs were isolated as previously described with minor modifications [18]. Blood was taken from adult, healthy donors according to institutional guidelines and the Declaration of Helsinki, and the study was approved by our ethics committee. Peripheral blood was drawn in 1/10 of Aster-Jandl anticoagulant. Apyrase (0.2 U/mL) was added to the blood before centrifugation (20 min at 280× *g*). Platelet-rich plasma (PRP) was diluted 1:1 with CGS buffer (120 mM of NaCl, 12.9 mM of trisodium citrate, 30 mM of d-glucose, pH = 6.5) and centrifuged for 10 min at 80× *g*. PRP supernatant was taken and centrifuged for 10 min at 380× *g*. The pellet was resuspended in CGS buffer, 0.2 U/mL of apyrase was added, and the mixture was centrifuged for 10 min at 380× *g*. The pellet was resuspended in 1 mL of HEPES buffer (145 mM of NaCl, 5 mM of KCl, 1 mM of MgCl_2_, 10 mM of HEPES, 10 mM of d-glucose, pH = 7.4), and PLT count was adjusted to 3 × 10^8^/mL. PLTs were left to rest for 15 min before inhibitor treatment and/or spreading.

As indicated, PLTs were pretreated with 10 µM of OCLR inhibitor YU142670 (SML-1806, Sigma Aldrich, Taufkirchen, Germany) and 100 nM of PI4KIIIα inhibitor GSK-A1 (SYN-1219-M005, Adipogen, Liestal, Switzerland) for 15 min. Then, PLTs were allowed to spread on glass coverslips in the presence or absence of the inhibitors in HEPES buffer for 45 min. After spreading, the cells were fixed and stained for PI4P or PI(4,5)P_2_, and actin.

### 2.3. HEK293T and BALB3T3 Cells

HEK293T cells and BALB3T3 cells were grown in DMEM (Dulbecco’s modified Eagle’s medium, Pan Biotech, Aidenbach, Germany) supplemented with 10% FBS (fetal bovine serum, Pan Biotech) and 1% penicillin/streptomycin (Lonza). Cells were seeded 24 h before treatment with 10 µM of OCRL inhibitor YU142670 (Sigma Aldrich) and 100 nM of PI4KIIIα inhibitor GSK-A1 (Vinci Biochem) for 1 h in full medium. Cells were then fixed and stained for PI4P and PI(4,5)P_2_, as described below.

### 2.4. Immunostaining and Confocal Fluorescence Microscopy

Initial immunostaining of intracellular (Golgi) and PM pools of PIs was performed as previously described [15]. Briefly, for the staining of the intracellular pool of PI(4,5)P_2_ and PI4P, all steps were performed at room temperature. Cells were fixed with 4% paraformaldehyde (PFA) in PBS for 15 min, rinsed with PBS containing 50 mM of NH_4_Cl, and permeabilized with 20 μM of digitonin in buffer A (20 mM of Pipes, pH 6.8, 137 mM of NaCl, 2.7 mM of KCl) for 5 min. After blocking (5% goat serum in PBS with 50 mM NH_4_Cl) for 45 min, cells were labeled with primary antibodies diluted 1:100 (anti-PI4P, anti-PI(4,5)P_2_), phalloidin-AF488 and DAPI, washed with buffer A, stained with secondary antibody (1:500), post-fixed for 5 min in 2% PFA, and mounted.

For the staining of the PM pool of phosphoinositides PI(4,5)P_2_ and PI4P, cells were fixed with 4% PFA in PBS with 0.2% glutaraldehyde (GA) for 15 min and rinsed with PBS containing 50 mM of NH_4_Cl. All subsequent steps were performed on ice with all pre-chilled solutions. Cells were blocked and permeabilized with 0.5% saponin (in buffer A containing 5% goat serum and 50 mM of NH_4_Cl) for 45 min, as previously described [15]. Optimization of PM staining protocol in cell lines and activated PLTs included permeabilization with 0.1, 0.5, or 0.8% saponin (in buffer A) for 1, 5, 30, 45 min or 1 h. Then, cells were labeled with primary antibodies diluted 1:100 (anti-PI4P, anti-PI(4,5)P_2_), and with phalloidin and DAPI, washed with buffer A, stained with secondary antibody (1:500), post-fixed for 10 min in 2% PFA on ice and additional 5 min on room temperature, and mounted.

Confocal images were obtained using an LSM880 confocal microscope (Carl Zeiss) equipped with an Argon-laser multiline (458/488/514 nm), HeNe laser (543 nm and 633 nm). The objective used was a Plan-Apochromat 63×/1.40 oil DIC III (Carl Zeiss). Fluorescence intensities were measured using Fiji ImageJ software [19]. For fluorescence intensity measurements, imaging conditions between different samples were kept constant. Fluorescence intensity was always expressed over the area (size) of cells.

### 2.5. Statistical Analysis

All experiments were performed at least in triplicate, and data are represented as mean ± standard error of the mean (SEM). Data were analyzed by ANOVA using Prism software (GraphPad). Differences were considered significant when *p* values were <0.05 (* *p* < 0.05; ** *p* < 0.01; *** *p* < 0.001; **** *p* < 0.0001).

## 3. Results

### 3.1. PI(4,5)P_2_ and PI4P Localize at Different Cellular Compartments in HEK293T and BALB3T3 Cells

First, we wanted to determine the localization of PI(4,5)P_2_ and PI4P in different cell lines to confirm previously established protocols for the subcellular distribution of these lipids [15]. To achieve this, we used human embryonic kidney cells (HEK293T) and mouse fibroblasts (BALB3T3). As described in detail in Methods, for imaging of intracellular PIs, cells were stained at room temperature and permeabilized with 20 μM of digitonin, while for PM staining, cells were stained on ice with 0.5% saponin permeabilization.

**Figure 1:**
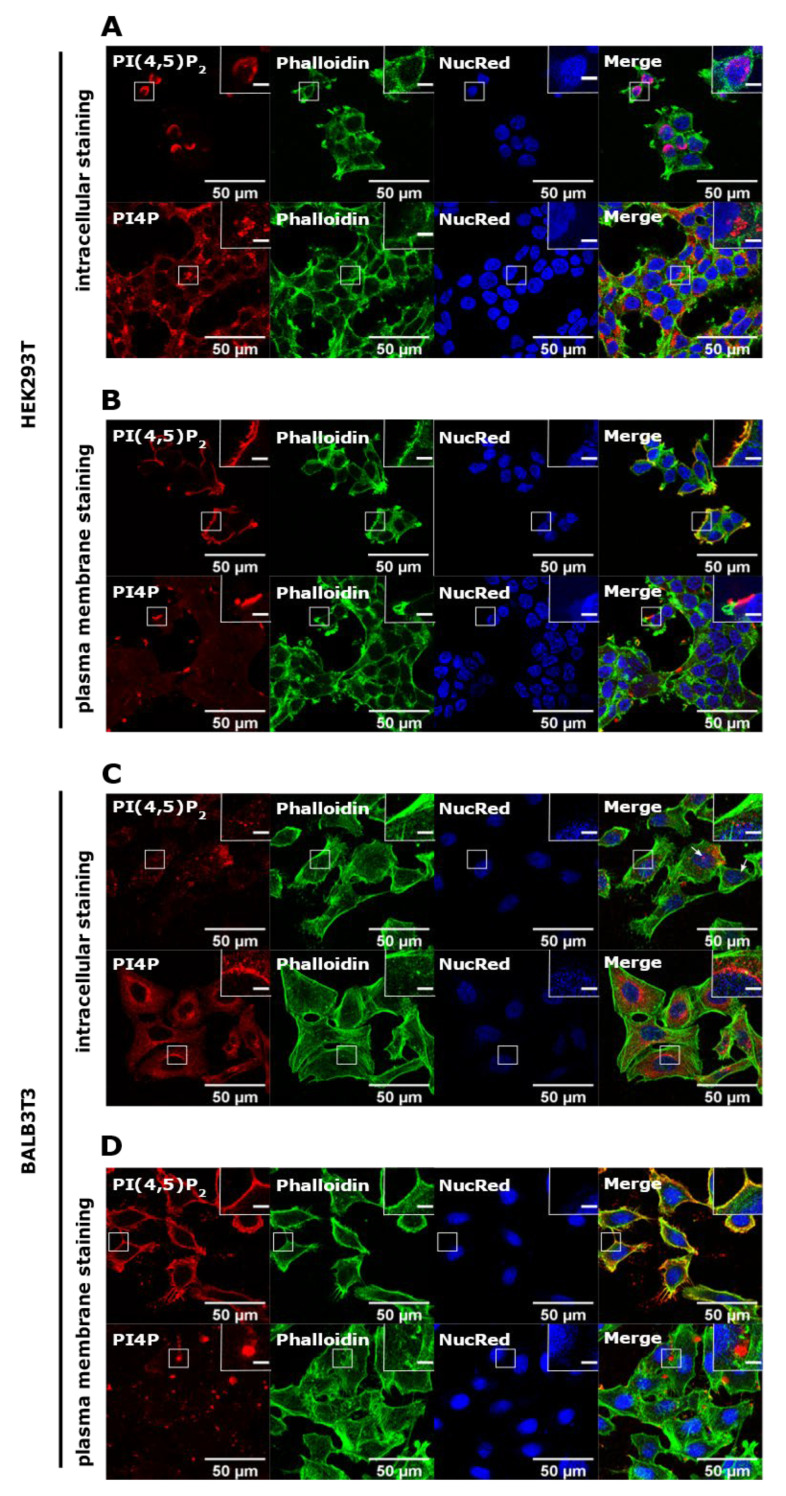
Intracellular and PM localization of PI(4,5)P_2_ and PI4P in HEK293T and BALB3T3 cell lines. HEK293T cells were fixed 24 h after seeding and were stained for (**A**) the intracellular pool or (**B**) the PM pool of PI(4,5)P_2_ and PI4P. The cells were co-stained for actin and the nucleus. BALB3T3 cells were fixed 24h after seeding and were stained for (**C**) the intracellular pool or (**D**) the PM pool of PI(4,5)P_2_ and PI4P. The cells were co-stained for actin and the nucleus. Representative images display a single confocal optical section. The scale bar of the images is 50 μm, while the scale bar of the inserts is 5 μm.

In HEK293T cells, the intracellular pool of PI(4,5)P_2_ localized to the parts of the nucleus of most of the cells (Figure 1A, upper panel). When the PM staining was performed, it displayed distinctive staining on the PM (Figure 1B, upper panel) consistent with the known PI(4,5)P_2_ localization [8,15,20]. On the other hand, the intracellular pool of PI4P localized mostly perinuclearly, where the Golgi apparatus can be found, as well as in vesicular structures throughout the cell (Figure 1A, lower panel). The PM pool of PI4P was displayed as bright dots on the PM (Figure 1B, lower panel), also consistent with the known PI4P localization [10,15].

In mouse BALB3T3 cells, intracellular PI(4,5)P_2_ mostly showed a fade dot-like pattern in the cytoplasm, while it only occasionally localized to the nucleus (Figure 1C, upper panel, arrows show nuclear localization). PM staining showed clearly that most of the lipid is at the PM (Figure 1D, upper panel). The intracellular pool of PI4P localized perinuclearly, as expected, and was shown in HEK293T cells (Figure 1C, lower panel), while it could also be found at the PM in the form of large bright dots (Figure 1D, lower panel). These data confirm known localizations of PI(4,5)P_2_ and PI4P, but also show differences in appearance and preferential localization regarding the cell type.

### 3.2. Resting and Activated Platelets Readily Stain for the Intracellular Pools but Not PM Pools of PI(4,5)P_2_ and PI4P

**Figure 2:**
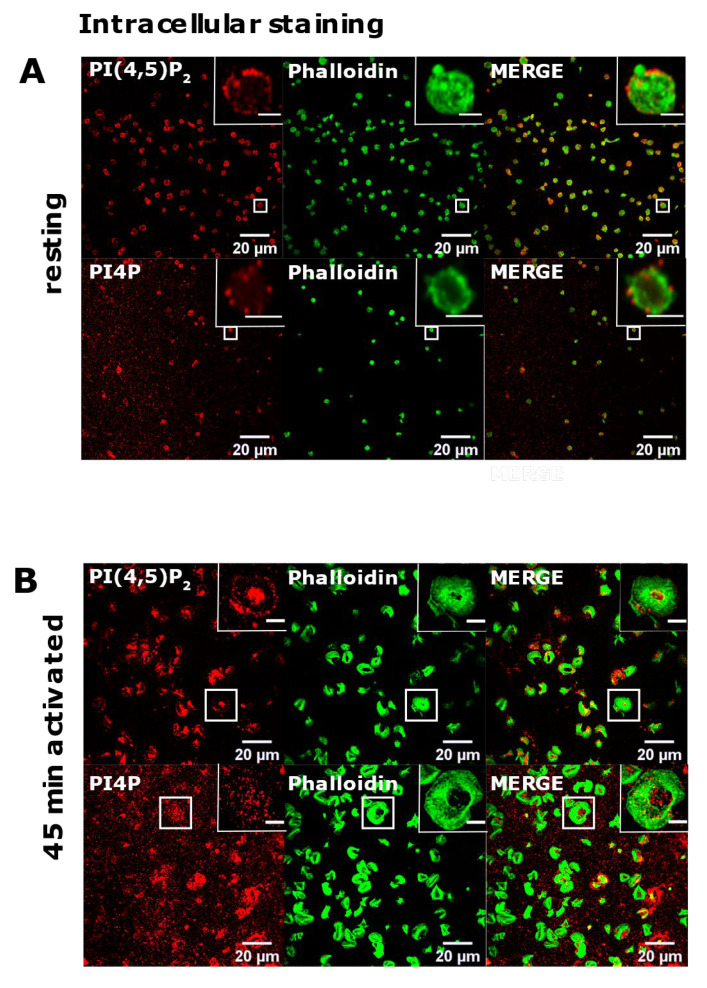
The intracellular localization of PI(4,5)P_2_ and PI4P in resting and activated PLTs. PLTs were isolated from human peripheral blood, (**A**) fixed immediately or (**B**) spread on glass for 45 min stained for the intracellular pools of PI(4,5)P_2_ and PI4P, co-stained for actin, and imaged with a confocal microscope. Representative images display a single confocal optical section. The scale bar of the images is 20 μm, while the scale bar of the inserts is 2 μm for resting, and 5 μm for activated PLTs.

Since we successfully reproduced the detection of diverse pools of PI(4,5)P_2_ and PI4P in two distinct cell lines, we next analyzed their localization in resting and activated PLTs isolated from human peripheral blood. Firstly, we examined the intracellular localization of PI(4,5)P_2_ and PI4P with the same protocol as for HEK293T and BALB cells [15] in resting PLTs that were fixed after isolation and resting period. In resting PLTs, PI(4,5)P_2_ displayed dot-like staining throughout the cell, but it was mostly localized at the PLT periphery (Figure 2A, upper panel). The vesicular pattern of staining for PI4P was to some extent different from PI(4,5)P_2_; the vesicular dots were bigger and scarcer (Figure 2A, lower panel). Although both lipids were found at the periphery of PLTs, they mostly localized just above the microtubular coil, and did not colocalize with the microtubules (Supplemental Figure S1, Supplementary Material).

Next, in the same manner [15], we examined the intracellular localization of PI(4,5)P_2_ and PI4P in PLTs that were spread and activated on glass for 45 min. Phalloidin staining revealed fully activated PLTs. Activated PLTs also clearly displayed intracellular pools of PI(4,5)P_2_ and PI4P. PI(4,5)P_2_ was localized mostly at the center of activated PLTs (Figure 2B, upper panel), while the staining of intracellular PI4P was less clear than PI(4,5)P_2_ with higher background but also showed an intracellular PI4P pool near the center of cells (Figure 2B, lower panel).

**Figure 3:**
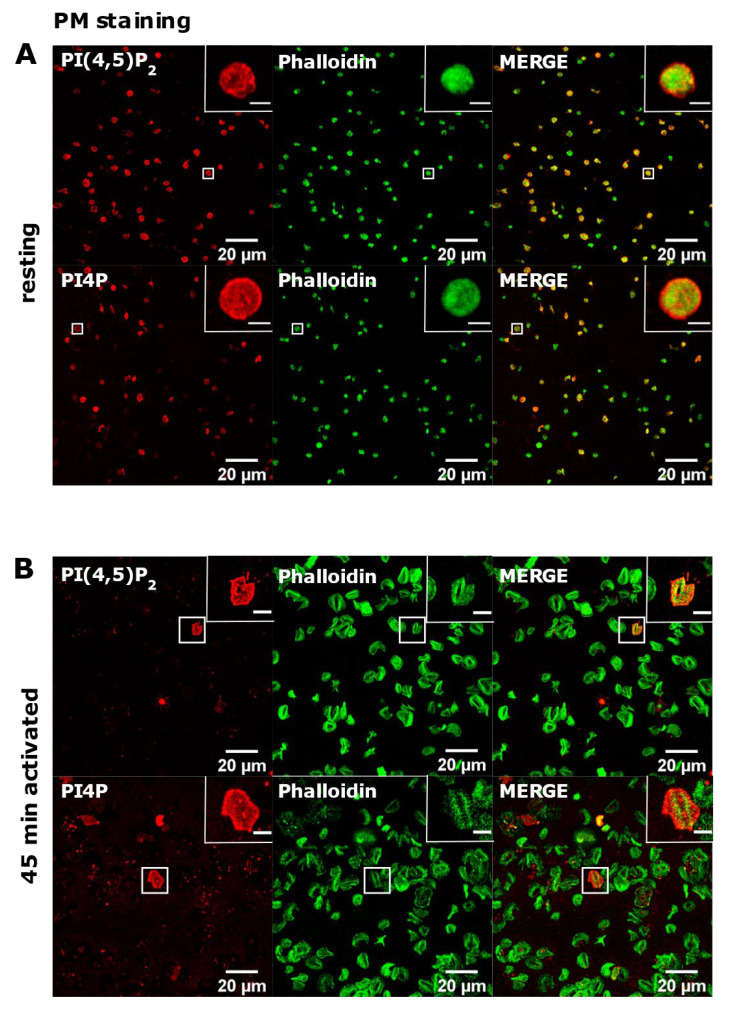
The plasma membrane localization of PI(4,5)P_2_ and PI4P in resting and activated PLTs. PLTs were isolated from human peripheral blood, (**A**) fixed immediately or (**B**) spread on glass for 45 min, fixed and stained for the plasma membrane pools of PI(4,5)P_2_ and PI4P, co-stained for actin, and imaged with a confocal microscope. Representative images display a single confocal optical section. The scale bar of the images is 20 μm, while the scale bar of the inserts is 2 μm for resting and 5 μm for activated PLTs.

Next, we investigated the PM pool of both lipids in PLTs. In resting PLTs, the staining of PI(4,5)P_2_ and PI4P following the established PM staining protocol [15] revealed that both lipids localized mostly to the edges of PLTs, indicative of PM (Figure 3A). However, a certain number of PLTs were void of any staining for both lipids. Moreover, in activated PLTs, only a few cells were stained for PI(4,5)P_2_ and PI4P, while most of the cells (>90%) displayed very dim staining or no staining at all (Figure 3B).

These data indicate that PI(4,5)P_2_ and PI4P in activated, and partially in resting PLTs, at the PM are not available for the antibodies or that lipids are being removed during the procedure by the current protocol.

### 3.3. PM Staining of PI(4,5)P_2_ and PI4P Can Be Improved by Decreasing the Permeabilization Time in Activated Platelets

**Figure 4:**
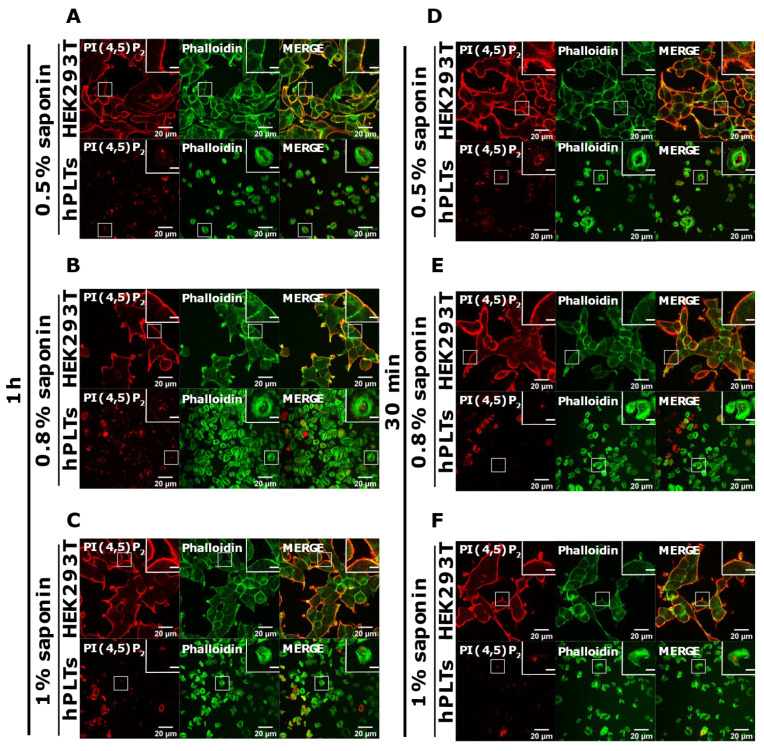

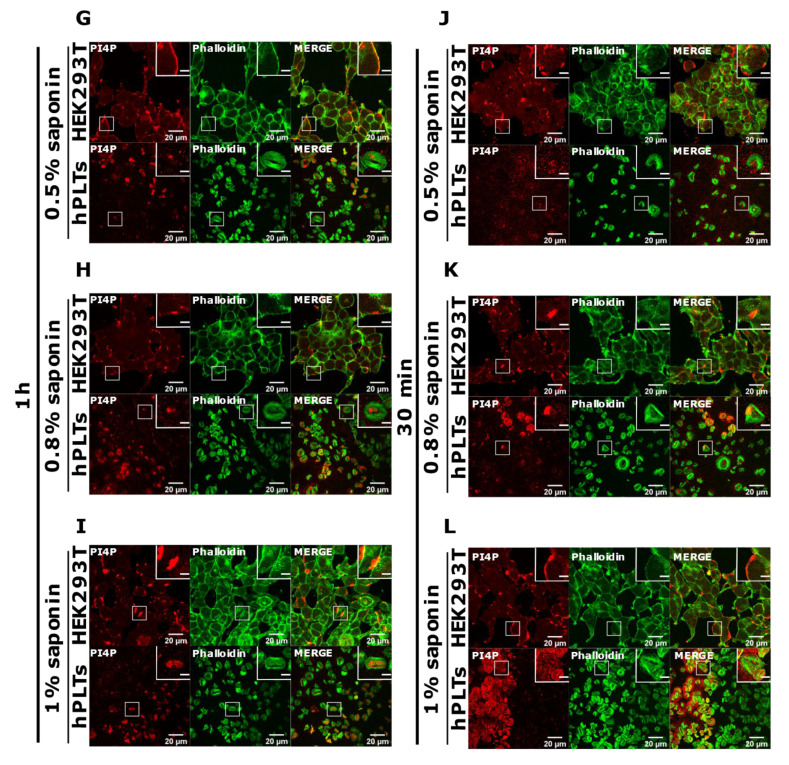
Modulation of the PM staining of PI(4,5)P_2_ and PI4P with 1 h and 30 min of permeabilization and different saponin concentrations. HEK293T cells were fixed 24 h after seeding and were stained for the PM pool of PI(4,5)P_2_ and PI4P, and co-stained for actin. PLTs were isolated from human peripheral blood, spread on glass for 45 min, fixed, stained for the PM pools of PI(4,5)P_2_ and PI4P, co-stained for actin, and imaged with a confocal microscope. (**A**–**C**) HEK293T cells and human PLTs were permeabilized for 1 h with (**A**,**G**) 0.5% saponin, (**B**,**H**) 0.8% saponin, and (**C**,**I**) 1% saponin and stained for (**A**–**C**) PI(4,5)P_2_ or (**G**–**I**) PI4P. (**A**–**C**) HEK293T cells and human PLTs were permeabilized for 30 min with (**D**,**J**) 0.5% saponin, (**E**,**K**) 0.8% saponin, and (**F**,**L**) 1% saponin and stained for (**D**–**F**) PI(4,5)P_2_ or (**J**–**L**) PI4P. Representative images display a single confocal optical section. The scale bar of the images is 20 μm, while the scale bar of the inserts is 5 μm.

PI(4,5)P_2_ is a major lipid at the PM where it serves as a source for downstream signaling events or modulates actin cytoskeleton polymerization, and PI4P is considered as its precursor [8]. As mentioned, processes are important also for PLT function, we attempted to optimize visualization of these lipids in activated PLTs. Since most of the PLTs were not stained by PM protocol, we first reasoned that they were not permeabilized enough, so we increased the permeabilization time from 45 min to one hour and increased the percentage of the permeabilizing agent, saponin, from 0.5 to 0.8% and 1%. As shown in Figure 4A–C,G–I, while HEK293T cells were readily stained for PI(4,5)P_2_ and PI4P at the PM in all tested conditions, the majority of PLTs still were not stained. Since the increased time of permeabilization and increased percentage of saponin did not result in better staining, we reasoned that PIs in PLTs are more sensitive to lipid extraction by permeabilizing agents than in HEK293T. Because of that, we decreased the permeabilization time to 30 min with 0.5%, 0.8%, and 1% saponin. As shown in Figure 4D–F,J–L, HEK293T cells were again stained in all tested conditions, while the majority of PLTs were not.

**Figure 5:**
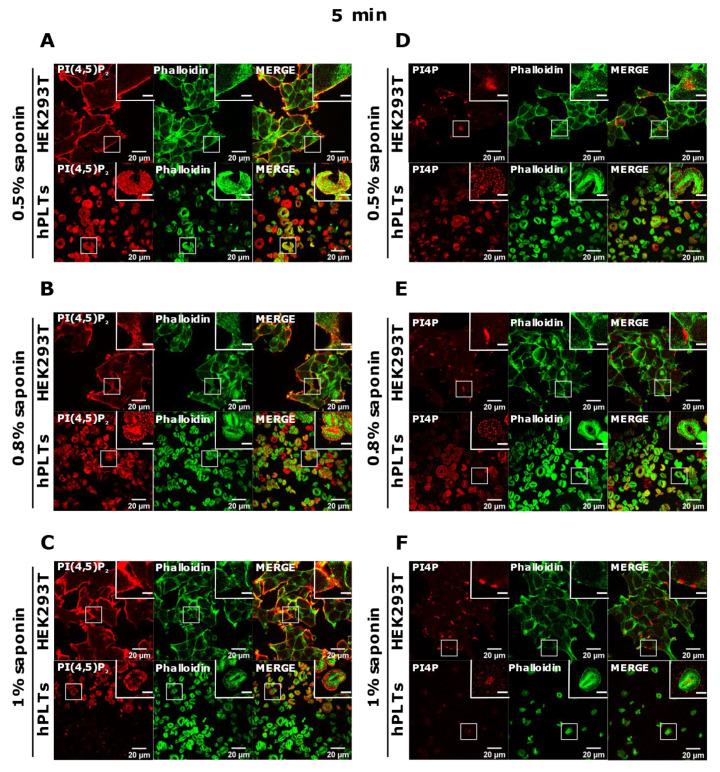
Modulation of the PM staining of PI(4,5)P_2_ and PI4P with 5 min of permeabilization and different saponin concentrations. HEK293T cells were fixed 24 h after seeding and were stained for the PM pool of PI(4,5)P_2_ and PI4P, and co-stained for actin. PLTs were isolated from human peripheral blood, spread on glass for 45 min, fixed, stained for the PM pools of PI(4,5)P_2_ and PI4P, co-stained for actin, and imaged with a confocal microscope. (**A**–**F**) HEK293T cells and human PLTs were permeabilized for 5 min with (**A**,**D**) 0.5% saponin, (**B**,**E**) 0.8% saponin, and (**C**,**F**) 1% saponin and stained for (**A**–**C**) PI(4,5)P_2_ or (**D**–**F**) PI4P. Representative images display a single confocal optical section. The scale bar of the images is 20 μm, while the scale bar of the inserts is 5 μm.

Since the staining was still not improved, we drastically decreased the permeabilization time to 5 min and used the three concentrations of saponin mentioned above. Again, HEK293T cells were stained in all tested conditions, and PLTs, in this case, displayed the best staining with 0.5% and 0.8% saponin for both PI(4,5)P_2_ and PI4P (Figure 5A,B,D,E). With the highest concentration of saponin (1%), fewer PLTs were stained, most probably because higher saponin concentration overly extracted lipids (Figure 5C,F). If 5 min of permeabilization was enough for PI(4,5)P_2_ and PI4P staining, we questioned whether even lower permeabilization time would be enough for successful staining and whether conditions without permeabilization could cause any staining. When permeabilizing the cells for one minute, most of the PLTs were stained for PI(4,5)P_2_ with 0.8% saponin (Supplemental Figure S2A,B, Supplementary Material), most (but not all) of the PLTs were stained for PI4P with 0.5% saponin (Supplemental Figure S2C,D), while the least PLTs were stained with 1% saponin for both lipids (data not shown). These data show that one minute is not enough for a successful PI(4,5)P_2_ staining with 0.5% saponin, and thus a higher concentration of 0.8% is needed. PI4P was visible in most of the cells permeabilized with 0.5% saponin; however, more cells were stained with shorter permeabilization (5 min). In conditions in which we did not use a permeabilizing agent, we observed low background or very low staining signals (Supplemental Figure S2E,F). The low staining could be due to fixation, which can in a very low amount permeabilize the cells. These results confirm that these lipids do not localize on the outer leaflet of the PM, but also show that antibodies do not bind unspecifically.

**Figure 6:**
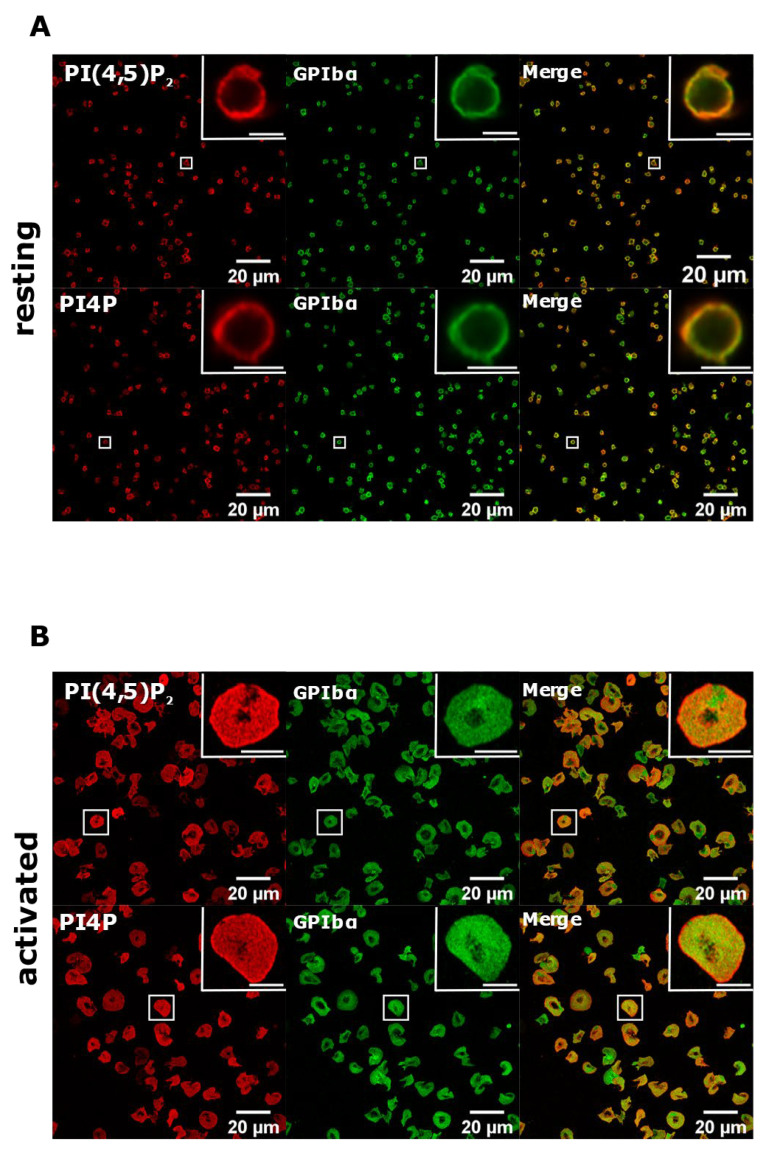
PM localization of PI(4,5)P_2_ and PI4P with the modified staining protocol. PLTs were isolated from human peripheral blood, (**A**) fixed or (**B**) spread on glass for 45 min, stained for the PM pools of PI(4,5)P_2_ and PI4P, co-stained for GPIbα, and imaged with a confocal microscope. Representative images display a single confocal optical section. The scale bar of the images is 20 μm, while the scale bar of the inserts is 2 μm for resting and 5 μm for activated PLTs.

To test whether with the improved staining protocol these lipids do localize to the PM, we co-stained resting and activated PLTs with glycoprotein Ibα (GPIbα), a PLT membrane marker [21]. As shown in Figure 6, both PI(4,5)P_2_ and PI4P clearly colocalized with GPIbα in resting PLTs, while the colocalization was partial in activated PLTs. We also analyzed whether PI(4,5)P_2_ and PI4P localize near the microtubular coil in resting PLTs with this protocol. As with the Golgi protocol, both lipids do not colocalize with the microtubular ring, but are in its close proximity (Supplemental Figure S3, Supplementary Material). Interestingly, the staining of the microtubules was fainter, suggesting that permeabilization with saponin for 5 min is not enough to clearly visualize the microtubules. Furthermore, the specificity of the antibodies against lipids was confirmed by staining with an IgM antibody followed by the secondary antibody and with only the secondary antibody (anti-IgM conjugated to Alexa Fluor 568). In both cases, staining gave no signal in resting (Supplemental Figure S4) or activated (Supplemental Figure S5, Supplementary Material) PLTs. Since the best staining of PI(4,5)P_2_ and PI4P at the PM was observed with 5 min of permeabilization with 0.5% saponin, we decided to use these conditions in our experiment.

### 3.4. Intracellular and PM PI(4,5)P_2_ and PI4P Can Be Modulated by OCRL and PI4KIIIα Inhibitors in Resting and Activated Platelets

**Figure 7:**
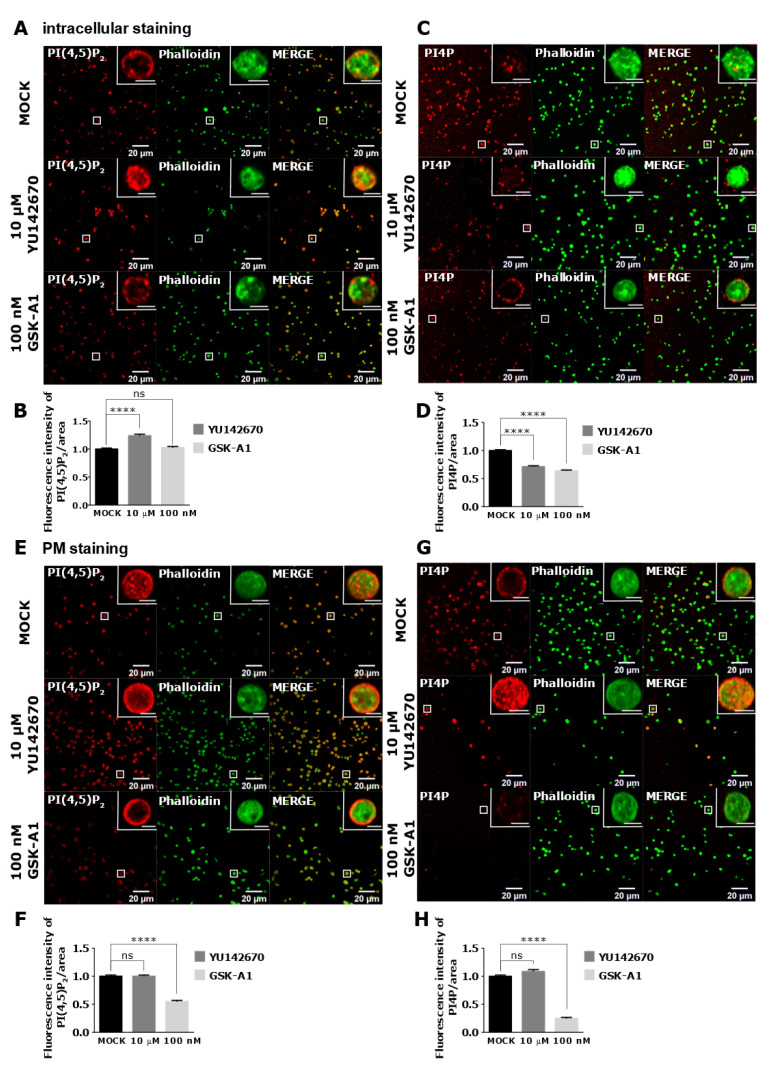
The intracellular and PM localization of PI(4,5)P_2_ and PI4P, visualized with the optimized protocol, in resting PLTs and their modulation by OCRL and PI4KIIIα inhibitors. PLTs were isolated from human peripheral blood, fixed, stained for the (**A**,**C**) intracellular or (**E**,**G**) PM pools of PI(4,5)P_2_ and PI4P, co-stained for actin, and imaged with a confocal microscope. The dephosphorylation of PI(4,5)P_2_ was inhibited by 10 µM OCRL inhibitor YU142670 and the production of PI4P was inhibited by 100 nM of PI4KIIIα inhibitor GSK-A1. Representative images display a single confocal optical section. The scale bar of the images is 20 μm, while the scale bar of the inserts is 2 μm. (**B**,**D**,**F**,**H**) Images were analyzed with Fiji ImageJ software to measure the mean fluorescence intensity of PI(4,5)P_2_ and PI4P. The graphs show the mean fluorescence intensity of PI(4,5)P_2_ and PI4P. Results in the graphs are presented as means, error bars denote ± SEM from 3 independent experiments. *, *p* < 0.05; **, *p* < 0.01; ***, *p* < 0.001; ****, *p* < 0.0001; ns, non-significant.

As staining of intracellular PI(4,5)P_2_ and PI4P was readily visible in resting PLTs, we decided to modulate their levels to examine their potential sources. We first inhibited OCRL phosphatase that dephosphorylates PI(4,5)P_2_ on position five of the inositol ring, resulting in the formation of PI4P [5]. The inhibition of the OCRL phosphatase with 10 µM of YU142670 inhibitor for 15 min resulted in a significant increase of PI(4,5)P_2_ (Figure 7A,B) and a significant decrease of PI4P levels compared to the control, as expected (Figure 7C,D). These results suggest that the anti-PI(4,5)P_2_ and anti-PI4P antibodies are specific for visualizing these two lipids in PLTs and that OCRL regulates a significant amount of PI(4,5)P_2_ levels that can be detected by immunocytochemistry. Results also suggest that approximately 30% of PI4P is formed by PI(4,5)P_2_ cleavage in resting PLTs intracellularly.

PI4KIIIα is a kinase that produces PI4P from PI mostly at the PM [22]. Human PLT proteome data revealed that PLTs express high levels of PI4KIIIα (1.800 copies per PLT), and although other PI4Ks were not found [23], transcriptomic data indicate a potential presence of other isoforms [24]. When PI4KIIIα is inhibited with its specific inhibitor GSK-A1 [25] (100 nM, 15 min), there was a decrease of ~36% in intracellular PI4P levels in resting PLTs (Figure 7C lower panel and D), suggesting that PI4KIIIα significantly contributes to the production of the steady-state intracellular pool of PI4P in resting PLTs. Interestingly, the inhibition of PI4KIIIα did not result in a significant decrease of the intracellular pool of PI(4,5)P_2_ (Figure 7A lower panel and B), suggesting that in conditions of acute inhibition of PI4KIIIα, its product PI4P is not a major source of intracellular PI(4,5)P_2_ in resting PLTs.

Next, we wanted to compare how inhibition of OCRL and PI4KIIIα affects PM levels of PI(4,5)P_2_ and PI4P in resting PLTs if a modified PM staining protocol was used. In resting conditions, all PLTs showed PM staining of both lipids (Figure 7E,G upper panels). However, inhibition of OCRL did not result in a change in PI(4,5)P_2_ or PI4P levels (Figure 7F,H). These data suggest that OCRL mainly affects the intracellular pool of PI(4,5)P_2_ and not the pool on the PM in resting PLTs. On the other hand, PI4KIIIα inhibitor resulted in a significant decrease of PI4P by 75% (Figure 7G,H) and a significant decrease of PI(4,5)P_2_ levels by 45% compared to the control (Figure 7E,F). These data suggest that PI4KIIIα is the main source of PI4P at the PM and that a significant portion of PI4P that is produced by PI4KIIIα at the PM serves as a substrate for PI(4,5)P_2_ in resting PLTs.

**Figure 8:**
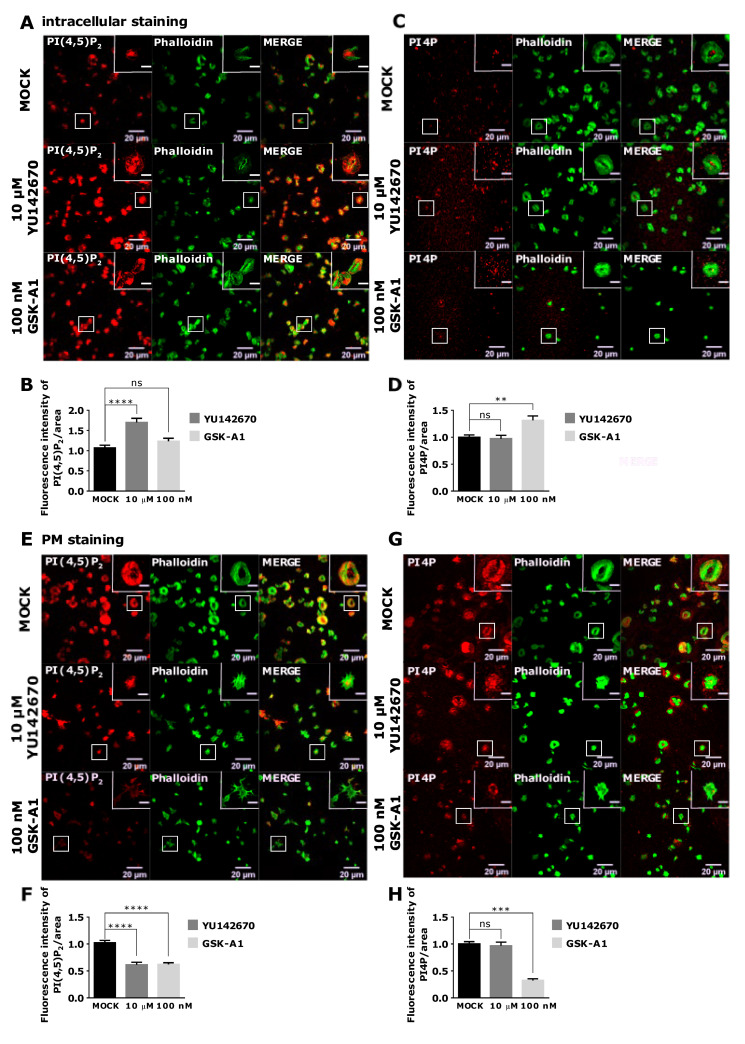
The intracellular and PM localization of PI(4,5)P_2_ and PI4P, visualized with the optimized protocol, in activated PLTs and their modulation by OCRL and PI4KIIIα inhibitors. PLTs were isolated from human peripheral blood, fixed, stained for the (**A**–**D**) intracellular or (E-H) PM pools of PI(4,5)P_2_ and PI4P, co-stained for actin, and imaged with a confocal microscope. The dephosphorylation of PI(4,5)P_2_ was inhibited by 10 µM of OCRL inhibitor YU142670, and the production of PI4P was inhibited by 100 nM of PI4KIIIα inhibitor GSK-A1. Representative images display a single confocal optical section. The scale bar of the images is 20 μm, while the scale bar of the inserts is 5 μm. (**B**,**D**,**F**,**H**) Images were analyzed with Fiji ImageJ software to measure the mean fluorescence intensity of PI(4,5)P_2_ and PI4P. The graphs show the mean fluorescence intensity of PI4P and PI(4,5)P_2_. Results in the graphs are presented as means, and error bars denote ± SEM from 3 independent experiments. *, *p* < 0.05; **, *p* < 0.01; ***, *p* < 0.001; ****, *p* < 0.0001; ns, non-significant.

Next, we analyzed intracellular levels of lipids in activated PLTs. As in resting, in activated PLTs, the inhibition of OCRL (10 µM YU142670) resulted in a significant increase of intracellular PI(4,5)P_2_ levels compared to the control (Figure 8A,B). However, OCRL inhibition, in this case, did not change PI4P levels (Figure 8C,D). Since activated PLTs release granules for which vesicular trafficking is extremely important, the potential decrease of PI4P production by PI(4,5)P_2_ cleavage could upregulate PI4 kinases to produce more PI4P and compensate its levels when OCRL is inhibited. However, the most surprising result was the increase of intracellular PI4P with no change of PI(4,5)P_2_ levels when PI4KIIIα was inhibited with 100 nM of GSK-A1 (Figure 8A–D, respectively). These results could be explained by the effect of depriving PI4P at the PM, as shown below so that PLTs need to compensate by increased intracellular production perhaps from other PI4K isoforms.

In activated PLTs, PI(4,5)P_2_ and PI4P localize to the PM (modified protocol, Figure 5A,D and Figure 8E,G). The inhibition of OCRL led to a significant decrease of PI(4,5)P_2_ levels compared to the control (Figure 8E,F) and did not change PI4P levels (Figure 8G,H). These results are opposite to the expected phenotype of OCRL inhibition. However, since the intracellular pool of PI(4,5)P_2_ is increased upon OCRL inhibition in resting and activated PLTs (Figure 7A,B and Figure 8A,B), these data suggest that OCRL is mainly responsible for the dephosphorylation of PI(4,5)P_2_ intracellularly and that low PM PI(4,5)P_2_ could be an indirect consequence of intracellular inhibition of OCRL in spread PLTs. Finally, the inhibition of PI4KIIIα resulted in a significant decrease of PI4P by 68% (Figure 8G,H) and a significant decrease of PI(4,5)P_2_ by 38% when compared to the control (Figure 8E,F). These data suggest that in spread PLTs, like in resting PLTs, PI4KIIIα produces the majority of PI4P at the PM, and a part of PI4P is a source for further production into PI(4,5)P_2_. It is important to emphasize that both inhibitors resulted in the partial inhibition of PLT spreading as observed by reduced PLT area, and this observation is a subject of our future investigation.

To further validate the staining protocol, we inhibited OCRL and PI4KIIIα in HEK239T cells. As shown in Supplemental Figure S6A,B (Supplementary Material), the inhibition of OCRL slightly decreased PI(4,5)P_2_ at the PM, with no change in PI4P levels which followed the results obtained in activated PLTs. On the other hand, the inhibition of PI4KIIIα significantly decreased PI4P with no change in PI(4,5)P_2_ levels. The drop of PI4P upon PI4KIIIα inhibition followed the results obtained in both resting and activated PLTs. Interestingly, no change in PI(4,5)P_2_ levels after PI4KIIIα inhibition in HEK293 cells is in contrast to the results obtained in PLTs, suggesting that PI4P produced by PI4KIIIα is not a major source of PI(4,5)P_2_ at the PM in these cells.

## 4. Discussion

The visualization of lipids with commercially available antibodies is a method that has been widely used to study the localization of different PIs. Immunocytochemical staining and analysis of PIs allow their visualization and detection on specific cellular compartments. However, caution should be taken when interpreting data because antibodies are much bigger than PIs and there is a high probability that one antibody recognizes two or more lipid molecules [26]. Nevertheless, the antibodies used in this study have been shown to be specific for PI(4,5)P_2_ and PI4P [27]. As observed in BALB3T3 cells, the PM is the main localization site of PI(4,5)P_2_, where it was shown to bind adaptor proteins for the formation of CCVs or promote actin reorganization for endocytosis [5]. Once the CCVs are formed, PI(4,5)P_2_ is internalized, and can be found on the vesicles that precede early endosomes. However, PI(4,5)P_2_ must be cleaved from these vesicles for normal endocytosis to occur [28]. The localization of PI(4,5)P_2_ in the nucleus, as in HEK293T cells, has been observed for a long time [29], and it was shown that there it promotes actin polymerization involved in transcription of RNA polymerase I, II, and III, and chromatin movement [30]. The fact that we observed PI(4,5)P_2_ in the nucleus of HEK293T cells, but not BALB3T3 cells, highlights the importance of choosing the right model for researching the role of PIs in specific cellular compartments. We also show that in HEK293T and BALB3T3 cell lines, PI4P localizes perinuclearly and in vesicles throughout the cell as well as at the PM. Indeed, the main site of localization for PI4P is the Golgi apparatus and vesicles that form from the Golgi and undergo anterograde trafficking towards the PM or retrograde trafficking towards late endosomes and lysosomes [11]. The PM localization is also well-known, but the role of PI4P there is still a matter of discussion as it is considered to be a main substrate of PI(4,5)P_2_ production [13,15].

Although the microscopic visualization of PIs with antibodies allows for the determination of the localization of distinct PIs in sub-cellular compartments, the method has several drawbacks. The fixation and permeabilization of cells could limit the accessibility of antibodies to PIs or change lipid composition. In addition, antibodies should recognize small differences of distinct PI species. Other methods have been developed and used to detect PIs in resting and activated platelets, such as mass spectrometry [31,32]. They accurately quantitatively measure PIs; however, they lack information on the spatial distribution of PIs as well as recognition of potential diverse cellular populations having different PI levels. In resting PLTs, only three PIs were detected by ion chromatography coupled to tandem mass spectrometry (IC-MS/MS), PI4P, PI(4,5)P_2_ (both present at high levels) and PI(3,4)P_2_ [32]. Collagen-related peptide (CRP) activation of PLTs caused the increase of all three PIs with the highest change in PI(3,4)P_2_; however, the kinases or phosphatases responsible for these changes were not studied. It would be interesting to see if inhibiting OCRL or PI4KIIIα in resting and activated PLTs would lead to similar PI4P and PI(4,5)P_2_ changes in IC-MS/MS as observed by immunofluorescence in our study.

While PM PI(4,5)P_2_ and PI4P were readily stained in resting platelets, very few cells were stained when PLTs were activated. Only a drastic decrease of permeabilization time, from 45 to 5 min with saponin, led to the PI staining of activated PLTs. Saponin, a plant glycoside used in the staining of the PM lipid pool, acts by selectively removing cholesterol, and making reversible holes in the membrane that are approximately 100 Å wide. Therefore, saponin is the mildest agent for permeabilization, and it is not an effective permeabilizing agent for cholesterol-poor membranes [17]. PLTs contain cholesterol-rich lipid rafts that are crucial for PLT function. It is possible that in activated PLTs, PI(4,5)P_2_ is more exposed to the effect of saponin. Lipid rafts have been shown to regulate PI(4,5)P_2_ in other cells [33]. Then, the increase of the amount of the permeabilizing agent perhaps could remove more lipids and, consequently, fewer PLTs are stained. On the other hand, digitonin is a saponin extracted from foxglove and, although also being a mild detergent, it forms permanent pores in the membranes by replacing cholesterol-lipid contacts in the membrane, but it can also solubilize cholesterol-free membranes [16]. Since resting PLTs do not excrete any granules and do not undergo shape change, membranes may be more resistant to the extraction of lipids. On the other hand, during PLT activation and shape change, the lipid composition of the PM could be more sensitive to lipid extraction because the membrane surface increases, filopodia and lamellipodia are formed, and granules are secreted. The exact cause of why PI(4,5)P_2_ and PI4P are more difficult to detect with the same procedure in activated PLTs remains to be determined. It could be that activation of PLTs causes redistribution of PIs and recruitment of effector proteins, and that some of the PIs are exposed and extracted by prolonged permeabilization. Less plausible is that PI epitopes for antibody binding remain hidden because of additional binding of effector proteins in spread PLTs since modified protocol finally detected PIs in PLTs. Another possibility is that antibodies do not efficiently detect PIs with different fatty-acyl residues, or they get extracted by permeabilization. Indeed, an interesting recent study using high-performance liquid chromatography–mass spectrometry (HPLC-MS) showed that activation of PLTs causes an increase in different fatty-acyl chain profiles of PIs, although the method could not distinguish between different regioisomers [31]. Nevertheless, the crucial finding was that both thrombin and CRP induced a strong increase in monophosphorylated and bisphosphorylated PIs that are less abundant fatty-acid species in resting, but increase up to 10-fold in activated, PLTs (e.g., in activated PLTs increase in C34:0, C34:2, C36:1, while major PIP_2_ species in resting PLTs are C38:4 and C38:3) [31].

In this study, we show that the intracellular pool of PI(4,5)P_2_ and PI4P can be modulated if we inhibit OCRL and PI4KIIIα. OCRL is a phosphatase that dephosphorylates PI(4,5)P_2_ on intracellular vesicles and produces PI4P [34]. On the other hand, PI4KIIIα is a kinase that specifically produces PI4P from PI mostly at the PM where it is transiently recruited [22]. The inhibition of OCRL in resting PLTs resulted in an increase of the intracellular pool of PI(4,5)P_2_ and a decrease of the intracellular pool of PI4P, while it did not change the PI(4,5)P_2_ and PI4P on the PM, suggesting that OCRL mainly functions on the intracellular pool of PI(4,5)P_2_. In agreement with the proposed site of major PI4KIIIα action being the PM [22], the inhibition of PI4KIIIα strongly decreases PI4P levels at the PM, and to a lower extent decreases PI(4,5)P_2_ levels. However, the inhibition of PI4KIIIα also resulted in a modest decrease of the intracellular PI4P pool, without the change of PI(4,5)P_2_ levels, in line with reported localization of PI4KIIIα at the ER and *cis*-Golgi in other cells [35]. Further studies on the contribution of other PI4K isoforms (located mainly at the Golgi) to the intracellular pools of PI4P in PLTs are underway.

During activation, PLTs release contents of dense and α-granules, like ADP, von Willebrand factor, or fibrinogen, which serve to recruit more PLTs at the site of injury and help to form a clot [36]. It has also been shown that Golgi subcompartments, packed in PLTs from MKs, contain glycosyltransferases and have a role in their secretion [37,38], which has been described as the non-conventional PLT protein secretion [39]. Interestingly, it has been shown in HeLa cells that the localization of Golgi glycosylation enzymes, glycan processing, and Golgi integrity depends on PI4P levels [12]. The role of PI4P in PLT secretion has not been investigated, but it is possible that this lipid has a role in the secretion of Golgi-derived vesicles and that the cells try to compensate the loss of PI4P when OCRL is inhibited by upregulating the production of PI4P via different routes, for example by PI4-kinases. Whether there is a change in the localization and function of the PI4- and PI(4,5)P_2_-producing kinases remains to be investigated. Furthermore, in activated PLTs, the inhibition of PI4KIIIα resulted in an increase of the intracellular pool of PI4P, a small but insignificant increase in PI(4,5)P_2_, and an expected decrease of the PM pool of both PI4P and PI(4,5)P_2_. It could be considered that the cells compensate for the loss of PI(4,5)P_2_ and PI4P on the PM by intracellular production. Indeed, it was shown in HeLa cells that the inhibition of PI4KIIIα lowers PI(4,5)P_2_ and PI4P at the PM and that it leads to an intracellular accumulation of PI(4,5)P_2_ and upregulation of PIPKIβ and PIPKIγ, two kinases that phosphorylate PI4P at position five, producing PI(4,5)P_2_ [22]. The authors concluded that the cells try to compensate for the loss of PI(4,5)P_2_ by the conversion of PI4P to PI(4,5)P_2_, but PI(4,5)P_2_ produced in this way is ectopically localized [22]. The same mechanism could occur in PLTs.

## 5. Conclusions

Finally, we conclude that, in addition to many differences in the role, structure, and function of resting and activated PLTs, there is also a difference in the composition and distribution of PI(4,5)P_2_ and PI4P in the PM that results in the more difficult detection of these lipids in activated than in resting PLTs. Nevertheless, by modulating permeabilization time and the amount of permeabilizing agent, we were able to optimize and reveal the localization of these two lipids.

## Data Availability

Data is contained within the article or Supplementary Material.

## Supplementary Materials

The following are available online at https://www.mdpi.com/article/10.3390/life11121331/s1, Figures S1–S6.
