# Supplementary material for: Imaging of Intracellular and Plasma Membrane Pools of PI(4,5)P2 and PI4P in Human Platelets
Source: Life (Basel). 2021 Dec 1;11(12):1331. doi: 10.3390/life11121331 (PMC8705196; doi:10.3390/life11121331)
Supplement: Supplementary file 1 [file life-11-01331-s001.zip › life-1479762-supplementary.pdf]

life

Article

Imaging of Intracellular and Plasma Membrane Pools of PI(4,5)P_2 and PI4P in Human Platelets

Ana Bura and Antonija Jurak Begonja *

Department of Biotechnology, University of Rijeka, 51000 Rijeka, Croatia; ana.bura@biotech.uniri.hr
* Correspondence: ajbegonja@biotech.uniri.hr; Tel.: +385-51-584-851

Citation: Bura, A.; Begonja, A.J. Imaging of Intracellular and Plasma Membrane Pools of PI(4,5)P2 and PI4P in Human Platelets. *Life* **2021**, *11*, 1331. https://doi.org/10.3390/life11121331

Academic Editor: Friedrich Jung

Received: 10 November 2021
Accepted: 28 November 2021
Published: 1 December 2021

Publisher's Note: MDPI stays neutral with regard to jurisdictional claims in published maps and institutional affiliations.

Copyright: © 2021 by the authors. Submitted for possible open access publication under the terms and conditions of the Creative Commons Attribution (CC BY) license (https://creativecommons.org/licenses/by/4.0/).

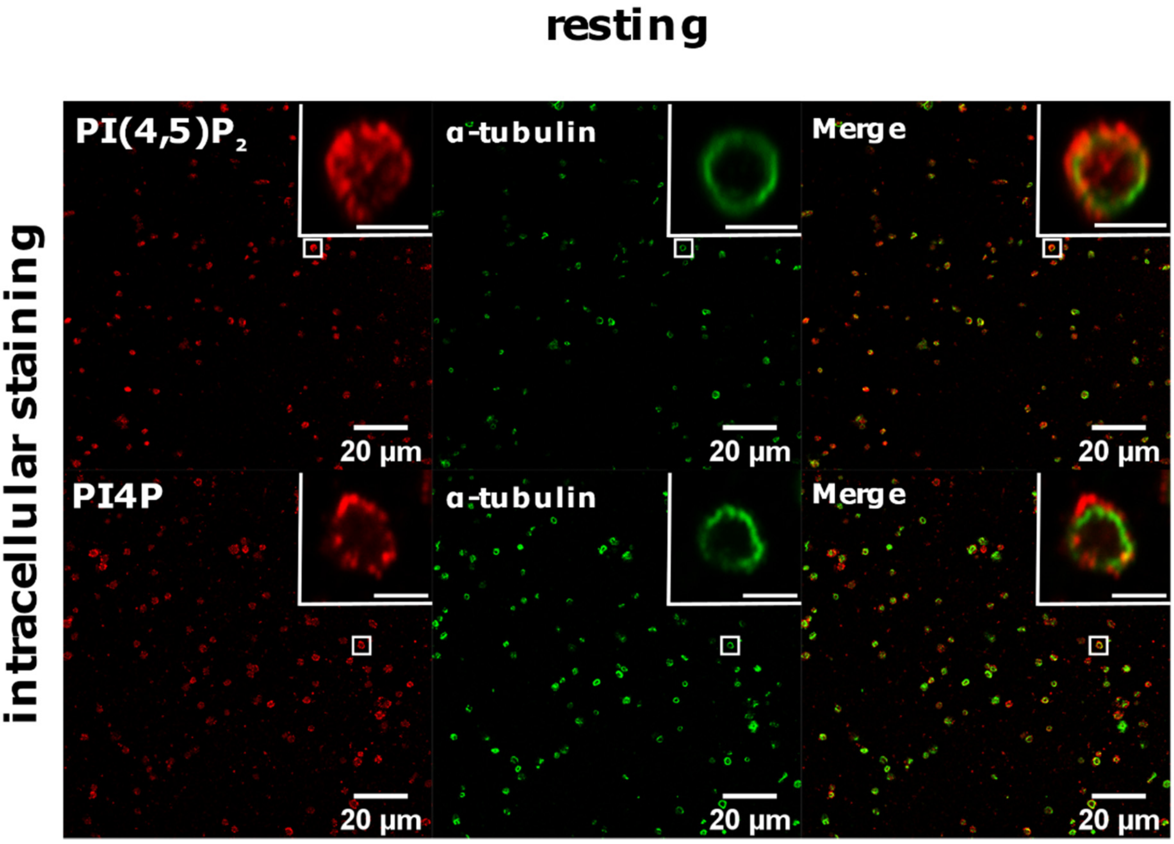

Supplemental material.

Supplemental Figure S1. PI(4,5)P2 and PI4P localize near the microtubular coil. PLTs were isolated from human peripheral blood, fixed immediately, stained for the intracellular pools of PI(4,5)P2 and PI4P, co-stained for α-tubulin, and imaged with a confocal microscope. Representative images display a single confocal optical section. The scale bar of the images is 20 μm while the scale bar of the inserts is 2 μm.

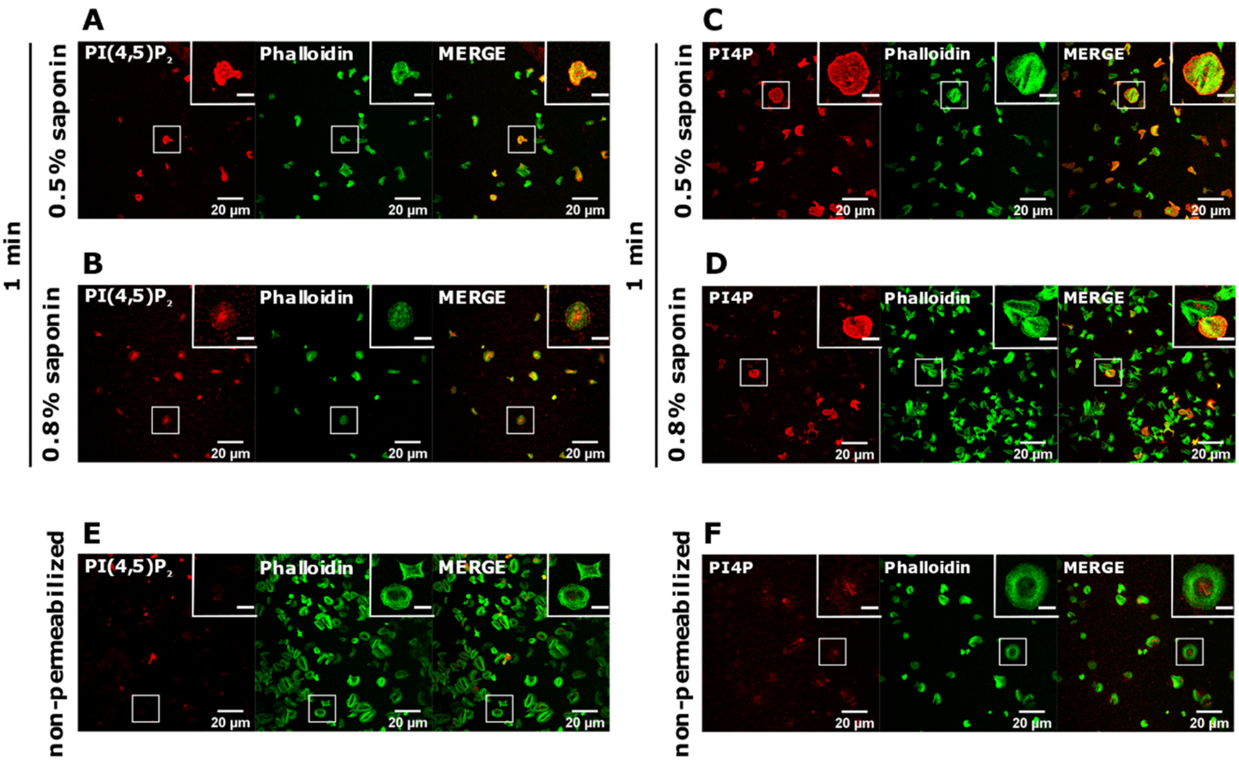

Supplemental Figure S2. Modulation of the PM staining of PI(4,5)P_2 and PI4P with 1-minute permeabilization and different saponin concentrations and staining of non-permeabilized cells. PLTs were isolated from human peripheral blood, spread on glass for 45 minutes, fixed, stained for the PM pools of PI(4,5)P_2 and PI4P, co-stained for actin, and imaged with a confocal microscope. (A-D) human PLTs were permeabilized for 1 minute with (A and C) 0.5% saponin, and (B and D) 0,8% saponin or (E-F) were not permeabilized and stained for (A, B, and E) PI(4,5)P_2 or (C, D and H) PI4P. Representative images display a single confocal optical section. The scale bar of the images is 20 μm while the scale bar of the inserts is 5 μm.

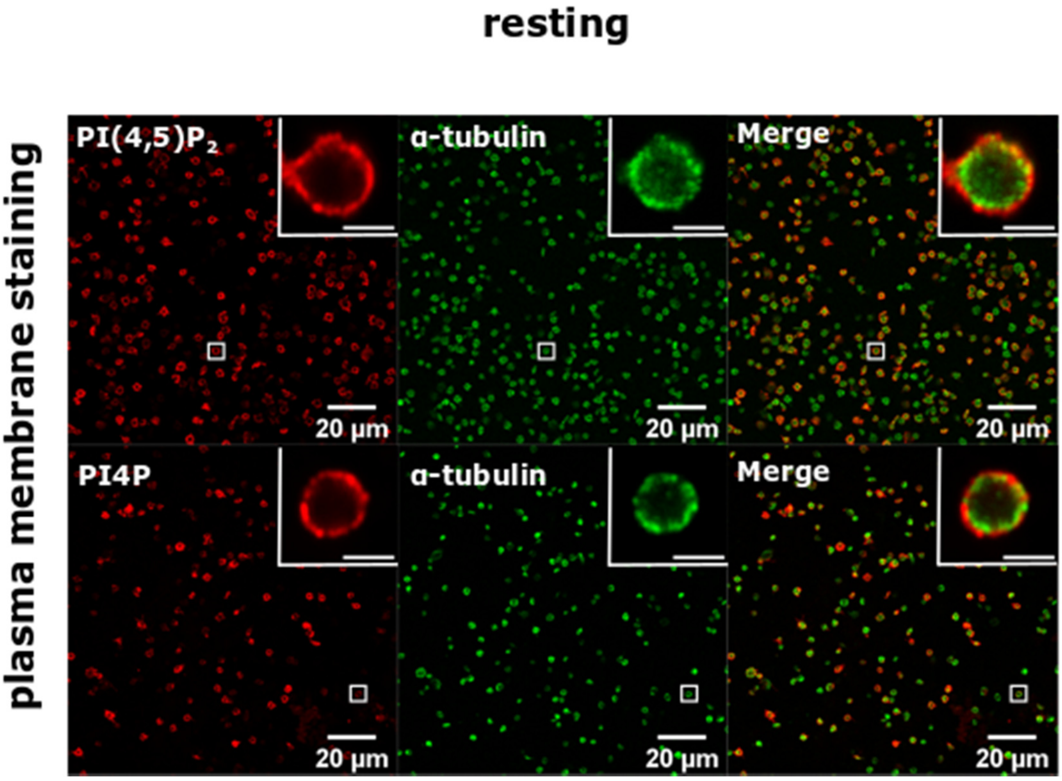

Supplemental Figure S3. The PM pool of PI(4,5)P2 and PI4P localizes near the microtubular coil. PLTs were isolated from human peripheral blood, fixed immediately, stained for the PM pools of PI(4,5)P2 and PI4P, co-stained for α-tubulin, and imaged with a confocal microscope. Representative images display a single confocal optical section. The scale bar of the images is 20 μm while the scale bar of the inserts is 2 μm.

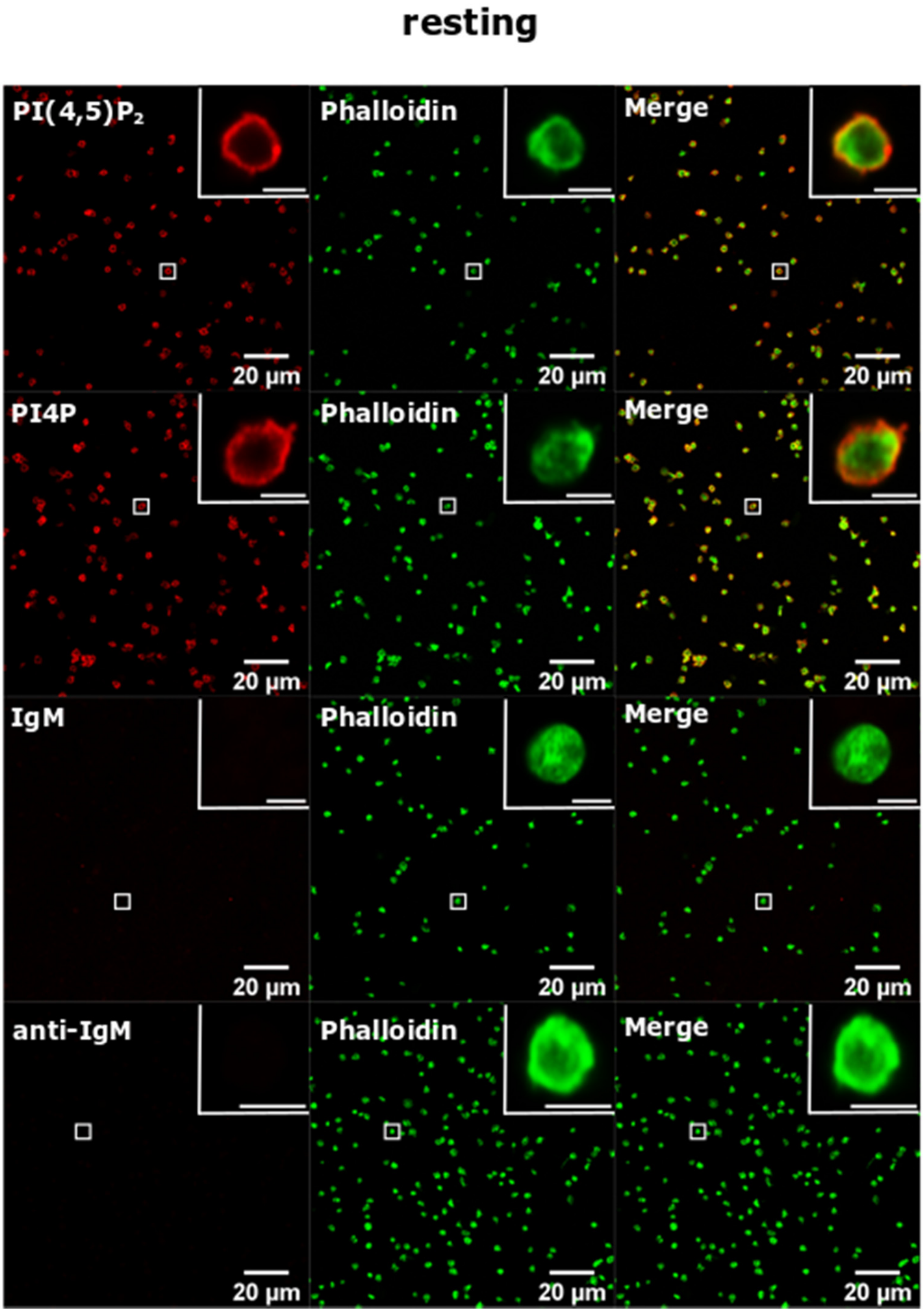

Supplemental Figure S4. Confirmation of the lipid antibodies specificity in resting PLTs. PLTs were isolated from human peripheral blood, fixed and stained against PI(4,5)P_2, PI4P, IgM or only anti-IgM conjugated to Alexa Fluor 568, co-stained for phalloidin, and imaged with a confocal microscope. Representative images display a single confocal optical section.The scale bar of the images is 20 µm while the scale bar of the inserts is 2 µm**.**

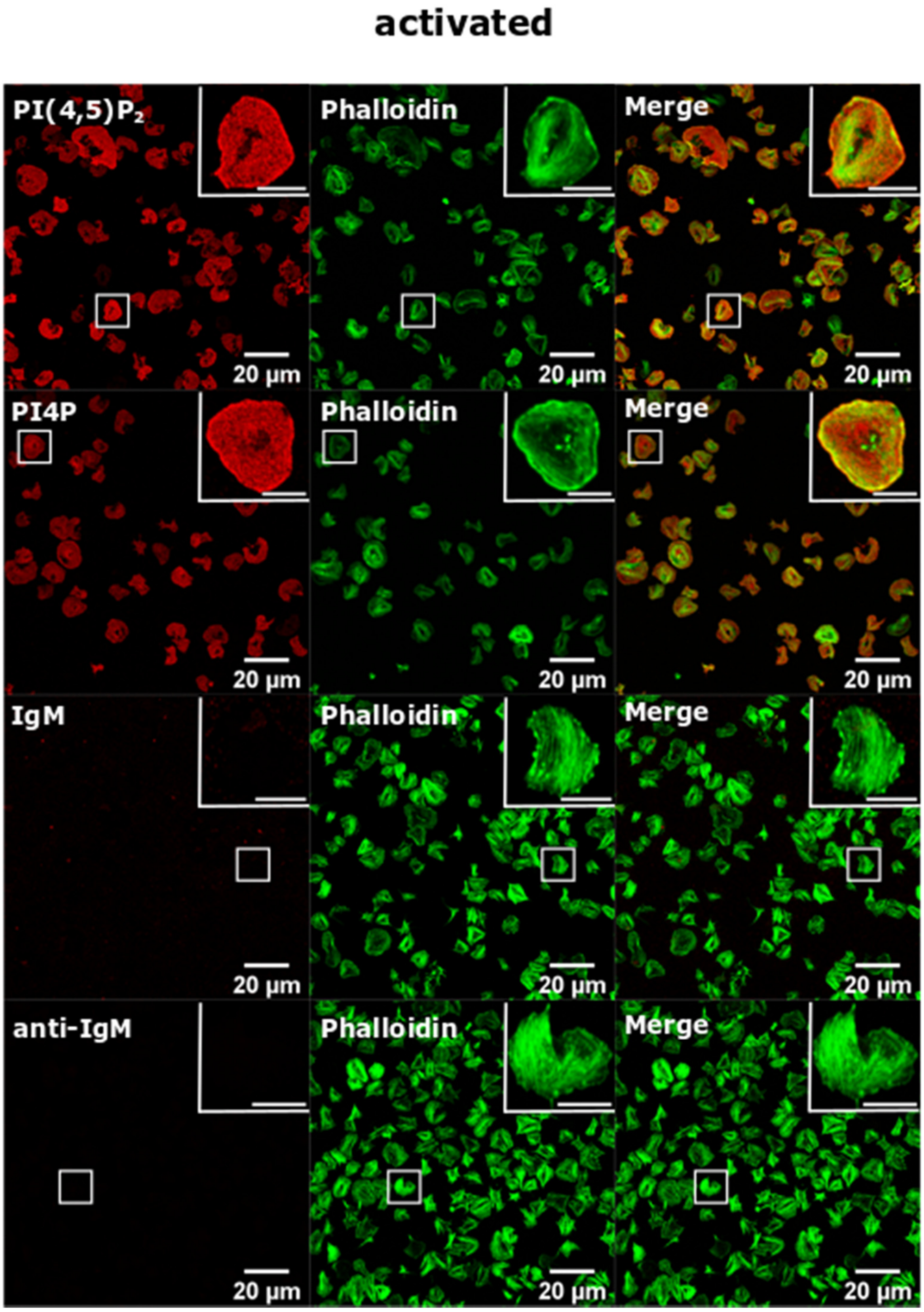

Supplemental Figure S5. Confirmation of the lipid antibodies specificity in activated PLTs. PLTs were isolated from human peripheral blood, spread on glass for 45 min, fixed and stained for PI(4,5)P2, PI4P, IgM or only anti-IgM conjugated with Alexa Fluor 568, co-stained for phalloidin, and imaged with a confocal microscope. Representative images display a single confocal optical section. The scale bar of the images is 20 µm while the scale bar of the inserts is 5 µm.

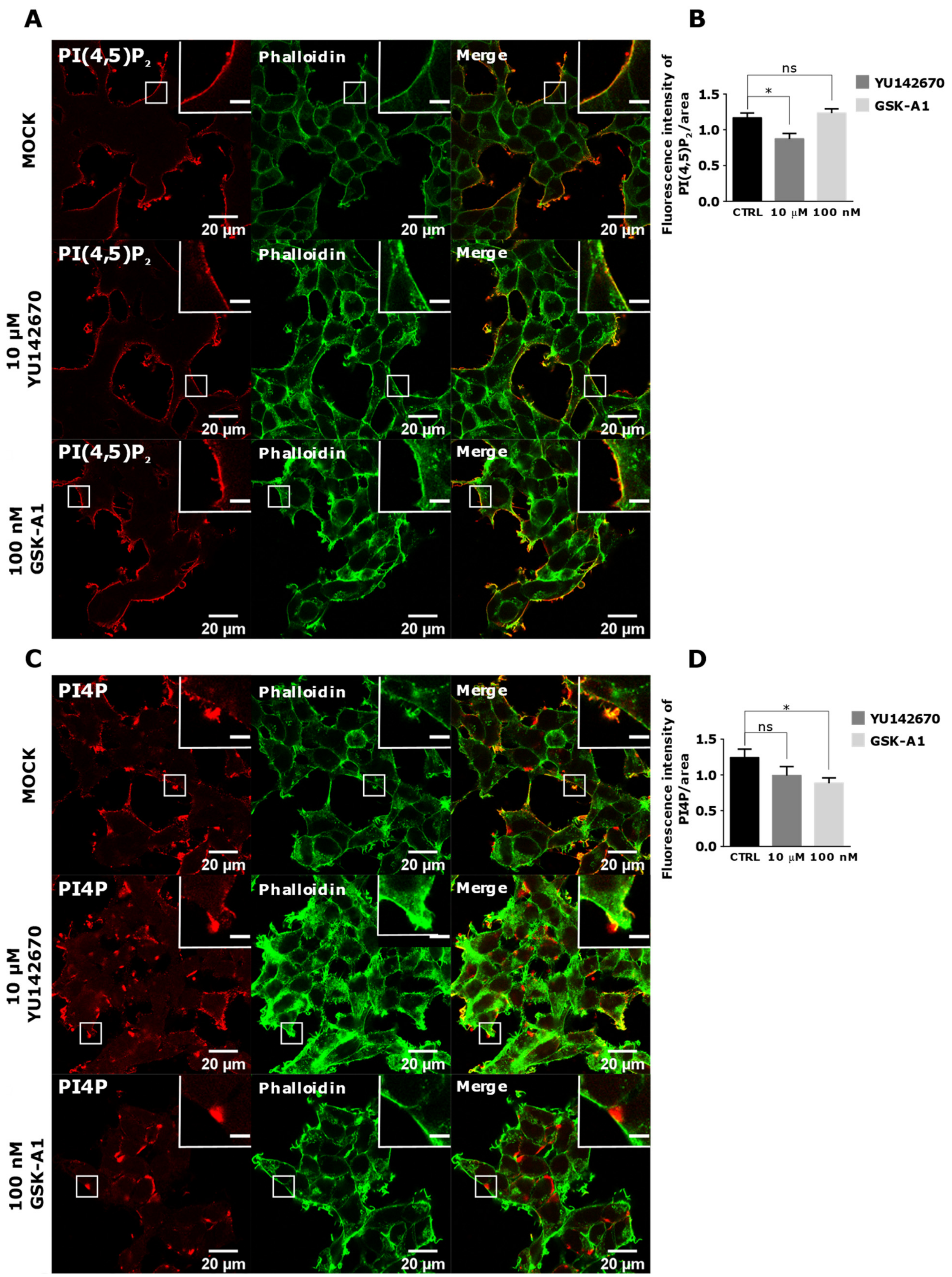

Supplemental Figure S6. The PM localization of PI(4,5)P2 and PI4P, visualized with the optimized protocol, in HEK293T cells and their modulation by OCRL and PI4KIIIα inhibitors. HEK293T cells were fixed 24h after seeding and were stained for the PM pool of (A) PI(4,5)P2 and (B) PI4P, co-stained for F-actin (phalloidin-Alexa Fluor 488), and imaged with a confocal microscope. The dephosphorylation of PI(4,5)P2 was inhibited by 10 µM OCRL inhibitor YU142670 and the production of PI4P was inhibited by 100 nM PI4KIIIα inhibitor GSK-A1 for 1 hour. Representative images display a single confocal optical section. The scale bar of the images is 20 µm while the scale bar of the inserts is 5 µm. (B and D) Images were

analyzed with Fiji ImageJ software to measure the mean fluorescence intensity of PI(4,5)P_2 and PI4P. The graphs show the mean fluorescence intensity of PI4P and PI(4,5)P_2. Results in the graphs are presented as means, error bars denote ± SEM from 2 independent experiments. *, $p < 0.05$; **, $p < 0.01$; ***, $p < 0.001$; ****, $p < 0.0001$; n.s., non-significant.
